# Do multidisciplinary ERAS protocols improve flap reconstruction outcomes? A structured review of efficacy, implementation challenges, and specialty-driven adaptations

**DOI:** 10.3389/fsurg.2026.1897143

**Published:** 2026-07-30

**Authors:** Meihui Xiao, Ximei Su, Ting Zhang

**Affiliations:** Clinical Nursing Teaching and Research Section, The Second Xiangya Hospital of Central South University, Changsha, Hunan, China

**Keywords:** enhanced recovery after surgery, ERAS, flap reconstruction, multidisciplinary care, reconstructive plastic surgery

## Abstract

**Background:**

Enhanced Recovery After Surgery (ERAS) pathways are increasingly used in reconstructive plastic surgery, but flap reconstruction presents specific challenges, including dependence on stable microvascular perfusion, procedure-specific immobilization, donor-site morbidity, and heterogeneous anatomic indications. Because available studies differ substantially in surgical setting, flap type, ERAS bundle composition, comparator care, and outcome reporting, this article is presented as a structured narrative synthesis rather than a *de novo* meta-analysis.

**Methods:**

PubMed, Embase, Web of Science, and Google Scholar were searched for studies and reviews published from 1997 to 14 June 2026 that addressed ERAS, fast-track, or accelerated recovery pathways in flap reconstruction or closely related reconstructive plastic surgery settings. The review question was defined using PICOS: adult or pediatric patients undergoing flap-based reconstruction; perioperative ERAS or accelerated recovery bundles; conventional perioperative care or pre-implementation practice; clinical, recovery, safety, and patient-centered outcomes; and randomized, prospective, retrospective, implementation, and systematic-review designs. Because ERAS bundles, flap types, and outcome definitions were clinically heterogeneous, no formal quantitative pooling was performed.

**Results:**

Across breast reconstruction, head and neck free-flap reconstruction, perineal or pelvic reconstruction, lower-limb reconstruction, and selected flap-based wound procedures, ERAS pathways were most consistently associated with shorter hospital stay, earlier mobilization or feeding, and reduced opioid exposure. Safety outcomes, including flap compromise, readmission, and return to theatre, were generally not worse in the cited studies, but certainty is limited by observational designs, single-center implementation studies, variable adherence, and inconsistent reporting. The components most relevant to flap surgery are preoperative optimization, multimodal analgesia, goal-directed fluid therapy, protocolized flap monitoring, early but protected mobilization, nutrition, and explicit nursing-clinician coordination.

**Conclusions:**

ERAS appears feasible and potentially beneficial in flap reconstruction when adapted to flap type, anatomic site, comorbidity burden, and reconstructive goals. The current evidence supports cautious implementation and local auditing rather than universal adoption of a single protocol. Future research should use plastic surgery-specific ERAS definitions, report study-level outcomes transparently, separate reconstructive from aesthetic indications where clinically appropriate, and prioritize long-term function, patient-reported outcomes, cost, and flap-specific safety endpoints.

## 1 Introduction

Flap surgery is a core reconstructive technique used to restore cutaneous, soft-tissue, and composite defects after trauma, oncologic resection, chronic ulcers, congenital conditions, and diabetes-related wounds (1, 2). Although flap reconstruction can restore form and function, perioperative recovery is complicated by the need to preserve tissue perfusion while controlling pain, edema, immobilization-related morbidity, donor-site recovery, nutrition, and patient-specific comorbidities (3–6).

ERAS is a multidisciplinary framework designed to reduce surgical stress, standardize perioperative care, and accelerate functional recovery. In colorectal, thoracic, and many oncologic specialties, ERAS pathways typically include preoperative counseling, nutrition, carbohydrate loading when appropriate, opioid-sparing analgesia, avoidance of excessive intravenous fluid, early feeding, and early mobilization. Flap reconstruction requires additional caution because early recovery goals must not compromise flap perfusion, vascular anastomoses, inset geometry, or donor-site integrity (4, 7–11).

For this reason, the question is not simply whether ERAS shortens length of stay. A clinically useful review must also ask whether recovery acceleration is achieved without increasing flap loss, vascular crisis, wound dehiscence, unplanned intervention, readmission, or functional compromise. The present review therefore focuses on efficacy, implementation requirements, safety signals, and the specialty-specific adaptations needed in flap reconstruction.

A further issue is the distinction between reconstructive and aesthetic plastic surgery. This distinction is useful, but it should not be treated as absolute. Some procedures, such as oncoplastic breast surgery or fat grafting after cancer treatment, include both reconstructive and aesthetic goals. In this review, the distinction is used pragmatically: reconstructive flap surgery is defined by tissue replacement for a defect or functional need, whereas aesthetic procedures prioritize appearance or contour in the absence of a major tissue-defect indication. Where procedures overlap, protocol decisions should be guided by operative magnitude, flap vascular dependence, donor-site morbidity, and functional goals rather than by label alone (12).

## 2 Methods

### 2.1 Review design

This article is a structured narrative review of ERAS in flap reconstruction. The cited literature includes different flap types, anatomic regions, ERAS bundle definitions, comparator groups, and outcome definitions, making a single pooled estimate clinically difficult to interpret without a complete study-level extraction database. Accordingly, no *de novo* formal meta-analysis was conducted.

### 2.2 Eligibility framework

The eligibility criteria were organized according to the PICOS framework summarized in Table 1.

**Table 1:** PICOS framework used to define the review question.

Domain	Definition Applied in This Review
Population	Patients undergoing flap-based reconstruction, including free, pedicled, local, or regional flaps in breast, head and neck, pelvic/perineal, lower-limb, hand, and selected wound reconstruction settings.
Intervention	A perioperative ERAS, fast-track, accelerated recovery, or enhanced recovery nursing pathway containing at least three coordinated components across preoperative, intraoperative, and postoperative phases.
Comparator	Conventional perioperative care, historical pre-ERAS practice, standard postoperative pathways, or comparator arms within randomized or prospective studies.
Outcomes	Length of stay, opioid use, time to mobilization or feeding, pain, complications, flap compromise, wound events, readmission, reoperation or unplanned intervention, functional recovery, patient-reported outcomes, and cost.
Study designs	Randomized trials, prospective and retrospective cohort studies, before-after implementation studies, quality-improvement studies, and systematic or scoping reviews. Case reports, *in vitro* studies, and purely aesthetic procedures without flap reconstruction were excluded from the main synthesis.

### 2.3 search strategy and selection process

A structured search was performed in PubMed, Embase, Web of Science, and Google Scholar for literature published from 1997 through 14 June 2026. The PubMed strategy was adapted for each database: ((flap reconstruction[Title/Abstract] OR free flap[Title/Abstract] OR skin flap surgery[Title/Abstract] OR flap-based reconstruction[Title/Abstract] OR reconstructive plastic surgery[MeSH Terms]) AND (enhanced recovery after surgery[Title/Abstract] OR ERAS[Title/Abstract] OR fast-track surgery[Title/Abstract] OR accelerated recovery[Title/Abstract] OR rapid recovery[Title/Abstract])). Reference lists of relevant reviews and original studies were also screened.

A formal PRISMA flow diagram was not generated because the available review records did not include the database exports, deduplication record, full-text screening log, and exclusion reasons needed to populate the diagram with verifiable counts. The search strategy, eligibility framework, and selection approach are therefore reported narratively in this structured review.

Two reviewers (Meihui Xiao and Ting Zhang) independently screened titles, abstracts, and full texts. Disagreements were resolved by discussion with a third reviewer (Ximei Su). Data were extracted by the same two reviewers using a standardized form that captured study design, sample size, surgical indication, flap type, ERAS components, comparator, follow-up, and reported outcomes. This wording replaces the prior inconsistent attribution of data extraction to a contributor not listed as an author.

### 2.4 Operational definition of ERAS exposure

ERAS was not treated as a single uniform intervention. A pathway was classified as ERAS or accelerated recovery only when it included at least three coordinated perioperative components and was explicitly implemented as a pathway rather than as an isolated intervention (Table 2).

**Table 2:** Minimum ERAS exposure criteria and flap-specific adaptations.

Phase	Core ERAS Components	Flap-Specific Considerations
Preoperative	Patient education, smoking cessation, nutrition screening or optimization, anemia or comorbidity optimization, expectation setting.	Identify vascular risk factors, malnutrition, diabetes, prior radiotherapy, and donor-site constraints; define functional goals and flap monitoring plan.
Intraoperative	Multimodal analgesia, opioid-sparing anesthesia, temperature control, antiemetic prophylaxis, goal-directed fluid therapy where appropriate.	Avoid both fluid overload and hypotension; preserve microvascular perfusion; tailor vasopressor and fluid decisions to free versus pedicled flap physiology.
Postoperative	Early feeding when safe, early mobilization, drain and catheter protocols, nausea and pain control, discharge criteria.	Mobilize early but protect inset and donor site; use standardized flap checks, Doppler or NIRS where available; coordinate nursing escalation for perfusion concerns.
Discharge and follow-up	Objective discharge criteria, patient education, follow-up pathway, complication surveillance.	Confirm flap stability, patient ability to recognize warning signs, wound-care capacity, and access to rapid review if perfusion or wound concerns occur.

### 2.5 Synthesis and quality assessment

No *de novo* pooled effect estimate or forest plot is reported in this structured narrative review. Where prior systematic reviews or original studies reported quantitative effects, they are described as study-level or review-level findings and interpreted in relation to design, setting, and risk of bias. Randomized trials were considered using Cochrane risk-of-bias domains; observational and implementation studies were considered using selection, comparability, confounding, outcome measurement, and follow-up domains consistent with Newcastle-Ottawa or ROBINS-I principles. PRISMA 2020 was used as a reporting benchmark for transparent description of the review question, search strategy, eligibility criteria, and limitations (16). Certainty was judged narratively rather than through a formal GRADE evidence profile because the evidence base spans multiple surgical populations and ERAS definitions.

## 3 Evidence base and study-level findings

**Figure 1:**
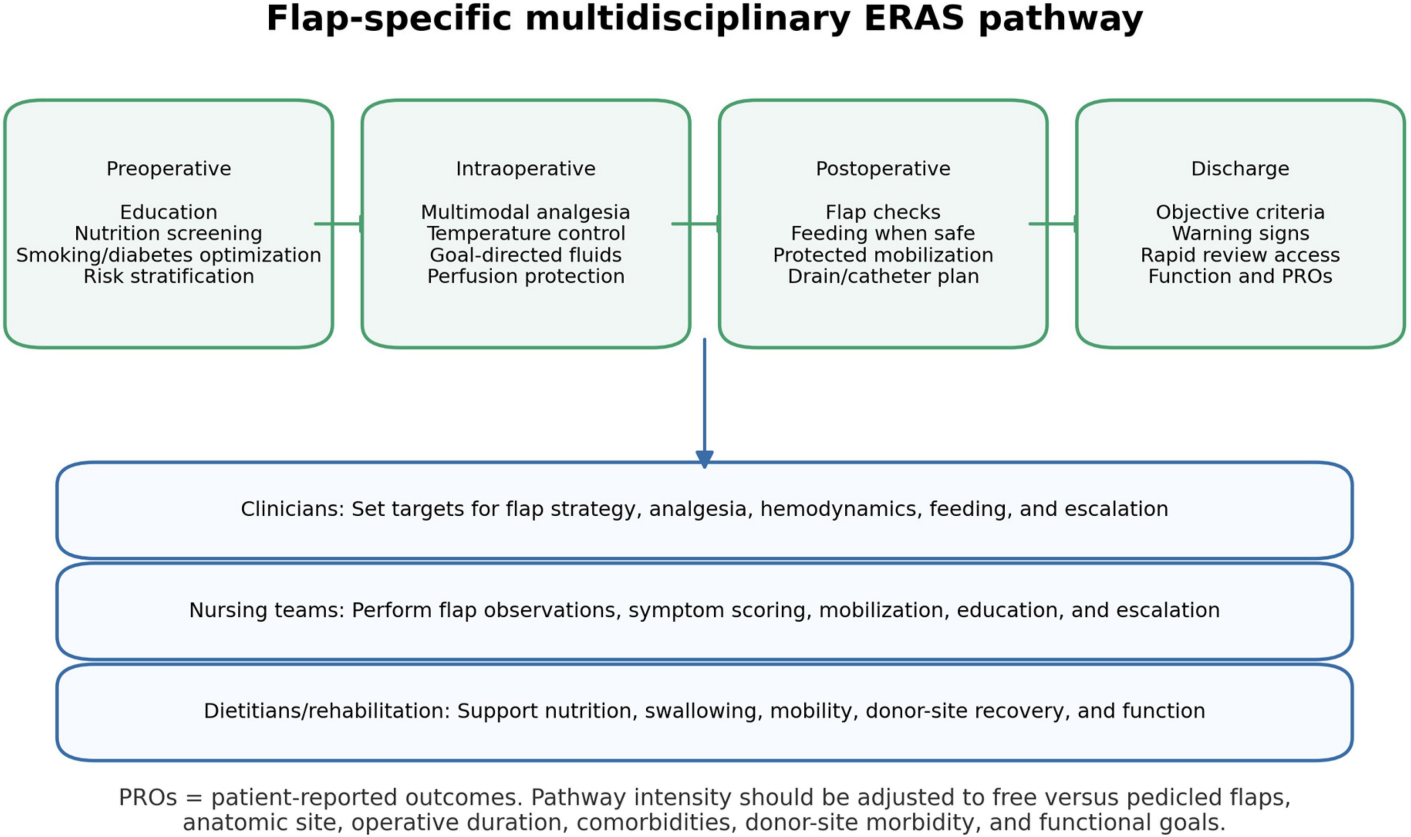
Flap-specific multidisciplinary ERAS pathway and role allocation across perioperative phases.

The evidence base includes prior systematic reviews, randomized or prospective studies in selected flap settings, retrospective cohort studies, and before-after ERAS implementation reports. The strongest and most consistent evidence relates to process and recovery outcomes such as length of stay, opioid use, time to mobilization, and time to feeding. Flap-specific safety outcomes are less consistently reported, and many studies are single-center or pathway-implementation designs (Table 3). The flap-specific multidisciplinary ERAS pathway is summarized in Figure 1.

**Table 3:** Evidence map of key studies and reviews used in the narrative synthesis.

Setting	Representative Evidence	ERAS Components Reported	Main Findings	Key Limitations
Microsurgical breast reconstruction	O'Neill et al. (4), Ochoa et al. (9), Gort et al. (10), Rendon et al. (22), Lombana et al. (12)	Preoperative education, multimodal analgesia, regional blocks, nausea prophylaxis, early feeding, early ambulation, discharge criteria.	Most studies reported shorter hospital stay and lower opioid exposure; available reports did not show a consistent increase in flap complications.	Mostly cohort or implementation designs; variable discharge culture; inconsistent reporting of readmission, flap take-back, and patient-reported outcomes.
Head and neck free-flap reconstruction	Bertelsen et al. (8), Twomey et al. (5), Nieminen et al. (13), Murr et al. (14)	Airway and feeding pathways, early mobilization, multimodal analgesia, standardized flap monitoring, multidisciplinary discharge planning.	Studies generally support feasibility, earlier mobilization or feeding, shorter ICU or hospital stay in selected pathways, and stable flap safety signals.	High clinical complexity; confounding by tumor stage, tracheostomy, nutrition, and adjuvant treatment; protocol adherence often incompletely reported.
Pelvic, perineal, and complex oncologic reconstruction	Pividori et al. (20) and related reconstruction literature	Preoperative imaging, wound care planning, nutrition, drain and catheter planning, protected mobilization.	Evidence supports the need for individualized pathways, particularly for wound healing, donor-site recovery, and voiding or mobility issues.	ERAS-specific comparative evidence remains sparse; many data are extrapolated from pelvic oncology and microsurgical reconstruction pathways.
Lower-limb, hand, and selected wound reconstruction	Tang et al. (3), Xu et al. (6), Lu et al. (15)	Enhanced recovery nursing, protected positioning, early but staged mobilization, pain control, wound monitoring.	Early mobilization and structured nursing pathways may shorten recovery milestones and improve patient experience when flap protection is maintained.	Anatomic and mechanical demands differ widely; small samples and mixed flap types limit generalizability.
Cross-setting reviews and methodological evidence	Tan et al. (11), Uhlman et al. (21), Page et al. (16)	Review-level synthesis and reporting standards.	Prior reviews support potential benefit of ERAS in flap-based reconstruction but highlight variable study quality and reporting.	Review conclusions depend on heterogeneous underlying studies and incomplete standardization of ERAS definitions.

## 4 Specialty-specific applications

### 4.1 Breast reconstruction

ERAS in microsurgical breast reconstruction is one of the best described flap-reconstruction settings. Common components include preoperative counseling, multimodal analgesia, regional anesthesia, nausea prevention, early oral intake, early ambulation, drain education, and structured discharge criteria (4, 9, 10, 12, 17, 18, 22). The most reproducible benefits are reduced hospital stay and opioid use. Interpretation should remain cautious, because discharge timing is influenced by institutional norms, payer systems, patient distance from care, and local nursing follow-up capacity.

Breast reconstruction also illustrates why the reconstructive-aesthetic distinction must be applied flexibly. Post-mastectomy DIEP reconstruction has flap-survival, donor-site, cancer-recovery, and psychosocial goals, whereas minor aesthetic fat transfer is less comparable in operative magnitude and vascular risk. Oncoplastic procedures may sit between these categories. Therefore, protocol intensity should be tied to flap vascular dependence, operative duration, donor-site morbidity, and functional recovery rather than a simple aesthetic versus reconstructive label. Short-stay ERAS approaches have also been described for selected locally advanced breast cancer patients undergoing immediate local thoracoabdominal advancement flap reconstruction, reinforcing the need to define the exact reconstructive context (19).

### 4.2 Head and neck reconstruction

Head and neck free-flap reconstruction requires pathways that integrate airway management, swallowing, nutrition, tracheostomy care, pulmonary risk reduction, flap monitoring, and early rehabilitation. Early mobilization after major head and neck surgery has been associated with improved recovery milestones, but patient readiness depends on hemodynamic stability, airway safety, donor-site limitations, and flap monitoring requirements (5, 8, 13, 14).

In this setting, nursing-clinician collaboration is not an optional add-on. Surgeons and anesthesiologists define the permissible range for hemodynamics, airway escalation, feeding, and flap monitoring, while nursing teams perform serial flap checks, document pain and mobilization tolerance, deliver oral-care and tracheostomy support, and trigger rapid review when perfusion, respiratory, or wound concerns emerge.

### 4.3 Perineal, pelvic, and complex oncologic reconstruction

Pelvic and perineal reconstruction often occurs in patients with previous radiotherapy, contamination risk, large dead space, urinary or bowel diversion, and prolonged wound healing. ERAS components should therefore be coordinated with pelvic oncology, urology, stoma care, wound care, and nutrition teams. The evidence base is less mature than in breast or head and neck reconstruction, and many recommendations remain extrapolated from pelvic oncology ERAS and microsurgical reconstruction principles (20).

Catheter management is a good example of the need for individualized protocols. Early catheter removal can reduce urinary tract infection and facilitate mobilization, but premature removal in long operations or pelvic flap reconstruction may cause urinary retention, pain, and pressure on reconstructed tissues. Protocols should be based on operative duration, urinary risk factors, flap location, ability to mobilize, and objective post-void assessment rather than a universal removal time.

### 4.4 Lower-limb, hand, and other flap-based reconstruction

Lower-limb and hand reconstruction create distinct challenges because flap protection may conflict with early ambulation or limb use. Nursing-led positioning, stepwise dangling or mobilization protocols, Doppler or clinical monitoring, pain control, and patient education are central to preventing venous congestion and mechanical stress. Studies of enhanced recovery nursing and selected flap procedures suggest faster recovery milestones, but the heterogeneity of defects, recipient vessels, fixation, and weight-bearing restrictions limits generalization (3, 6, 15).

## 5 Outcomes and interpretation

Given the heterogeneity of study designs, flap types, ERAS bundles, comparator groups, and outcome definitions, this review does not present *de novo* pooled effect estimates or aggregate sample sizes. The outcome synthesis below is qualitative and source-specific (Table 4).

**Table 4:** Outcome domains and strength of evidence in the revised synthesis.

Outcome Domain	Direction of Evidence	Interpretation for Practice
Length of stay	Most consistently reduced in breast and head and neck ERAS implementation studies.	Potentially useful endpoint, but must be interpreted with readmission, access to follow-up, and patient readiness.
Opioid use and pain	Frequently improved with multimodal analgesia and regional anesthesia pathways.	Strong rationale for routine inclusion, with attention to renal, hepatic, and bleeding risks.
Early mobilization or feeding	Often achieved earlier under protocolized care.	Should be staged and flap-specific; mobilization must not compress pedicles or compromise donor sites.
Flap compromise, return to theatre, or flap loss	Available studies generally do not show worse safety signals, but reporting is inconsistent.	Safety claims should be conservative and audited locally using standardized flap-specific endpoints.
Readmission and unplanned intervention	Reported inconsistently across studies.	Should be included in future ERAS studies to ensure shorter stay does not shift risk after discharge.
Long-term function and patient-reported outcomes	Underreported.	Future studies should include limb function, swallowing, quality of life, satisfaction, and cost.

## 6 Risk of bias and evidence certainty

Risk of bias is a major limitation of the current evidence. Many ERAS studies compare a new pathway with historical practice, which is vulnerable to secular trends, changes in surgical technique, discharge policy, and staffing. Randomized evidence is limited to selected settings and may not be powered for rare flap-specific safety outcomes. The certainty of conclusions should therefore be matched to the outcome: recovery-process outcomes are more consistent than flap-loss, reoperation, or long-term functional outcomes. This interpretation is consistent with recent methodological appraisal showing variable quality and reporting across plastic surgery ERAS studies (21).

**Figure 2:**
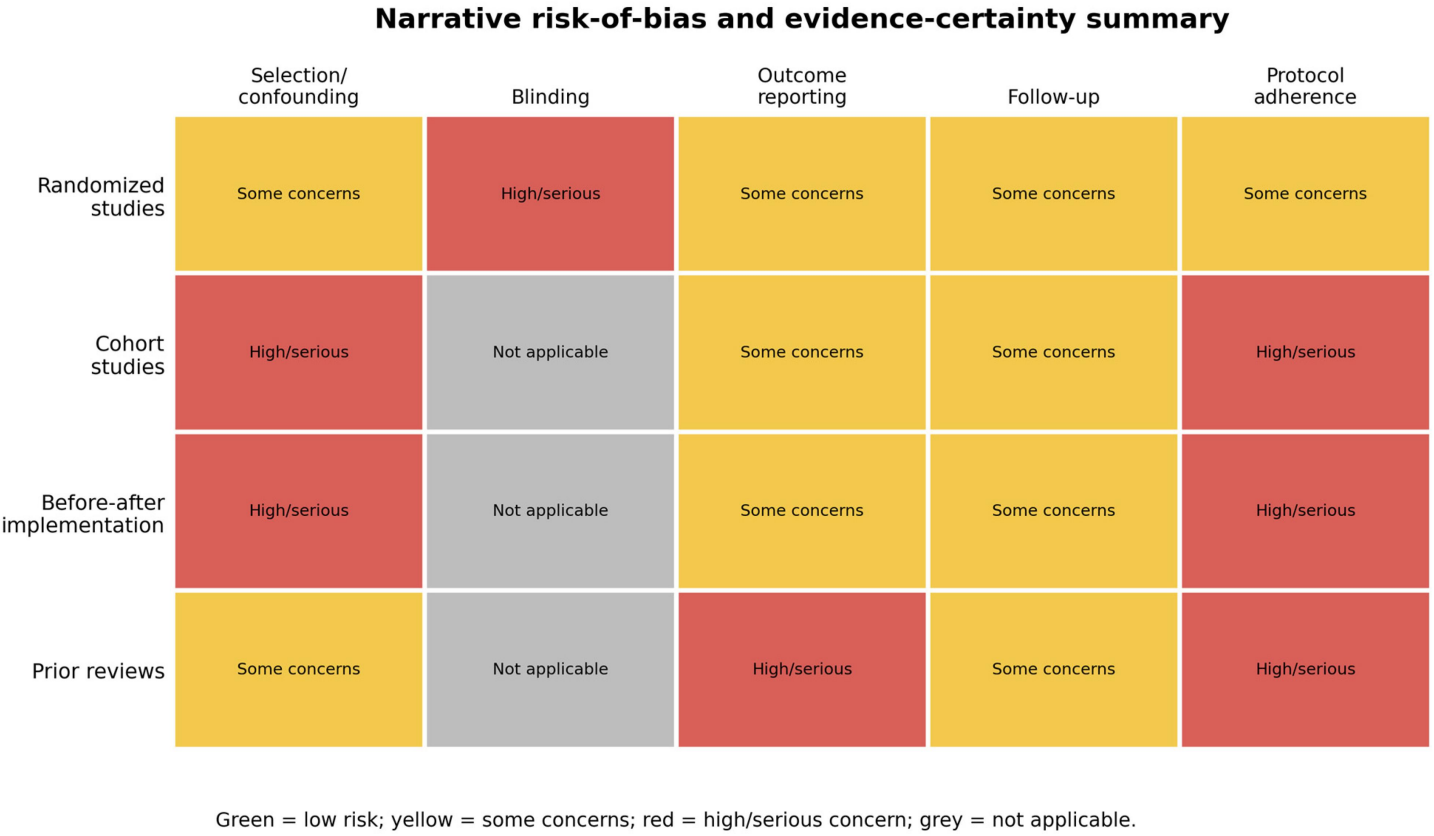
Narrative risk-of-bias and evidence-certainty summary by study design.

The visual summary presents the main risk-of-bias concerns narratively by evidence type. It is not a study-level Cochrane or ROBINS-I traffic-light plot; rather, it is a transparent visual summary aligned with the revised structured narrative design. The narrative risk-of-bias and certainty summary is shown in Figure 2 and detailed in Table 5.

**Table 5:** Narrative risk-of-bias and certainty summary.

Evidence Type	Main Bias Concerns	Certainty for ERAS Benefit
Randomized trials in selected flap-related settings	Limited blinding, single-procedure focus, small sample sizes for rare safety outcomes.	Moderate for short-term recovery endpoints; low to moderate for flap-specific safety endpoints.
Prospective or retrospective cohort studies	Selection bias, residual confounding, protocol-adherence variation, center effects.	Low to moderate, depending on adjustment and completeness of outcome reporting.
Before-after implementation studies	Secular trends, concurrent staffing or practice changes, Hawthorne effect.	Low for causal inference but useful for feasibility and implementation lessons.
Prior systematic reviews	Depend on heterogeneous underlying studies and inconsistent ERAS definitions.	Helpful for mapping evidence; less definitive for universal pooled effects.

## 7 Implementation challenges and practical solutions

### 7.1 Fluid management

Fluid management is central in flap reconstruction because both under-resuscitation and over-resuscitation can be harmful. Hypotension may threaten perfusion, whereas excessive crystalloid can contribute to tissue edema and venous congestion. Free flaps with microvascular anastomoses generally require closer hemodynamic attention than small local or pedicled flaps. ERAS pathways should specify target blood pressure ranges, monitoring frequency, escalation thresholds, and the role of vasopressors or goal-directed fluid therapy in local practice (7, 8).

### 7.2 Catheter, drain, and mobilization protocols

Removal of catheters and drains should be protocolized but not automatic. Earlier removal may reduce infection, pain, and immobility, but prolonged operations, pelvic reconstruction, urinary retention risk, or limited mobility may justify individualized timing. Mobilization protocols should be stepwise: sitting, dangling, standing, and walking should be advanced according to flap location, pedicle geometry, donor-site closure, pain, orthostatic symptoms, and nursing assessment.

### 7.3 Standardization and audit

The most practical next step is not a single universal ERAS pathway, but a standardized reporting framework. Each study and clinical pathway should report: patient population, flap type, reconstructive indication, ERAS components, adherence, comparator care, discharge criteria, length of stay, complications, flap compromise, readmission, unplanned intervention, and follow-up duration. Local audits should monitor whether shorter stays are accompanied by stable or improved safety endpoints.

## 8 Future directions

Future studies should define plastic surgery-specific ERAS algorithms that stratify by free versus pedicled flaps, anatomic site, operative duration, vascular risk, radiotherapy, diabetes, smoking, and donor-site morbidity. Multicenter prospective studies should use standardized outcomes and report adherence to each ERAS component.

Artificial intelligence and remote monitoring are promising but remain largely a research agenda in this field. Machine-learning models may eventually help predict flap risk or interpret continuous monitoring data, but current claims should be limited to feasibility and hypothesis generation unless supported by direct clinical validation (23, 24).

Long-term outcomes should receive greater attention. Short-term discharge success is incomplete without data on wound healing, function, pain, return to work, swallowing, limb mobility, donor-site morbidity, quality of life, aesthetic satisfaction where relevant, and cost.

## 9 Limitations

This review has several limitations. First, the evidence base is clinically heterogeneous, including multiple anatomic regions, flap types, indications, and ERAS bundle definitions. Second, many original studies are observational or before-after implementation studies, which limits causal inference. Third, publication bias is possible because successful ERAS programs may be more likely to be reported than neutral or difficult implementation experiences. Fourth, long-term functional, patient-reported, and cost outcomes remain underreported. Fifth, this revision does not present a *de novo* meta-analysis or formal PRISMA flow diagram because a complete study-level extraction file, database export, deduplication record, and full-text exclusion log were not available for independent verification. For future submission as a formal systematic review, the authors should provide the full search log, deduplication record, exclusion reasons, and study-level extraction table as supplementary material.

## 10 Conclusion

ERAS pathways in flap reconstruction are clinically plausible, feasible, and often associated with faster recovery milestones, especially shorter hospital stay, earlier feeding or mobilization, and lower opioid exposure. However, the strength of evidence varies by setting, and the current literature does not justify unsupported universal pooled estimates. ERAS should be implemented as a flap-specific, multidisciplinary pathway with explicit nursing-clinician roles, protected mobilization, careful fluid management, standardized monitoring, and local auditing of flap safety, readmission, and long-term functional outcomes.

## Data Availability

All data discussed in this review are available in the published articles cited in the reference list. No unpublished data were generated.
